# Evaluating the Remineralizing Effects of Calcium Sucrose Phosphate and Casein Phosphopeptide-Amorphous Calcium Phosphate Toothpastes on Artificial Carious Lesions Using Micro-computed Tomography: An In Vitro Investigation

**DOI:** 10.7759/cureus.67637

**Published:** 2024-08-23

**Authors:** Siddhant U Thorat, Nivethigaa Balakrishnan, Aravind Kumar Subramanian

**Affiliations:** 1 Orthodontics and Dentofacial Orthopaedics, Saveetha Dental College and Hospitals, Saveetha Institute of Medical and Technical Sciences, Saveetha University, Chennai, IND

**Keywords:** remineralisation, preventive, non-caries enamel lesions, demineralisation, dental

## Abstract

Background

White spot lesions (WSLs) are common early indicators of enamel demineralization, particularly in pediatric orthodontic patients. Effective remineralization of these lesions is crucial for preventing further dental decay. This study aimed to evaluate the three-dimensional remineralization efficacy of two commercial toothpastes, calcium sucrose phosphate (CaSP) and casein phosphopeptide-amorphous calcium phosphate (CPP-ACP), using micro-computed tomography (micro-CT).

Objectives

To compare the remineralization efficacy of CaSP and CPP-ACP on artificially created WSLs in human premolar enamel using micro-CT assessment.

Materials and methods

Freshly extracted, caries-free human premolars were used for the purpose of the study. Teeth with any defects, including caries, non-carious lesions, fractures, or hypocalcifications, were excluded. Teeth were stored in a saline solution with 0.1% thymol at +4°C until experimentation. Enamel slabs (N = 18) of 3 x 3 x 1.5 mm were prepared from the buccal surfaces of the premolars and polished to a mirror-like finish. The slabs were divided into three groups (n=6 each): control, CaSP, and CPP-ACP. Specimens were demineralized in a demineralization solution for 72 hours and then treated with the respective toothpastes or remineralizing solutions for 13 days. Micro-CT scanning was performed to assess changes in enamel volume and mineral density. Statistical analysis included Shapiro-Wilk tests, paired t-tests, and one-way ANOVA with post-hoc Tukey's HSD tests.

Results

Enamel volume changes were significant between groups (p<0.01), with the CaSP group showing the largest remineralization effect. Enamel mineral density changes were also significant (p=0.004), with the CPP-ACP group showing the greatest improvement in mineral density.

Conclusions

CaSP and CPP-ACP are both effective in remineralizing artificial enamel lesions. While CaSP shows comparable efficacy to CPP-ACP, further research is needed to confirm these findings in clinical settings. CaSP paste can be considered a viable, cost-effective alternative for enamel remineralization.

## Introduction

Human enamel, a remarkable and intricate tissue, forms the outermost layer of our teeth and plays a crucial role in maintaining their structural integrity and function. Enamel is renowned for its exceptional hardness, resistance to wear, and aesthetic properties. It has a hard surface with a pore-filled microstructure because of the spaces between prisms [1]. Maintaining dental health depends critically on the equilibrium between remineralization and demineralization in the oral cavity. Demineralization and remineralization are in balance, with a bias towards demineralization when the pH in the oral cavity falls below the crucial threshold of 5.5. This happens as a result of phosphate (PO43-) and calcium (Ca2+) ions being removed from the tooth's surface. Enamel is susceptible to a variety of carious and non-carious lesions despite its tough exterior. White spot lesions (WSLs), which are defined as opaque, chalky-white patches on the surface of the tooth, indicate the beginning of enamel demineralization and are frequently indicators of more advanced dental decay [2]. However, under ideal circumstances, it is possible to restore the enamel without invasive procedures and shift the equilibrium back in favour of remineralization.

Orthodontic therapy is renowned for its lengthy duration, with a typical fixed appliance treatment extending for approximately 24.9 months on average. Demineralization of the enamel surface near the bracket due to plaque accumulation occurs in many cases [3]. Two preferred locations for this accumulation include the areas around the tooth's cervical margins and beneath the bands, especially in regions where the cementing material has been washed away [4]. The composition of bacteria in dental plaque undergoes substantial alterations, with increased levels of acid-producing bacteria, especially *Streptococcus mutans* and *Lactobacilli* [5].

Various other modalities have been employed in the past to eradicate WSL during and after orthodontic treatment [6]. These include the use of fluoride products, remineralizing agents lasers, antiseptic treatments [7,8], probiotics, etc. during the course of orthodontic treatment [9-11]. Post-treatment modalities such as bleaching, microabrasion, and resin infiltration have also been employed [12].

A classic technique that is mostly used for clinical detection is visual assessment following air drying [13]. Visual assessment methods for detecting WSLs are limited by subjectivity and lack of sensitivity, often failing to identify early or subsurface demineralization. A number of other techniques have been developed in the past 10 years, including laser fluorescence, digital imaging with fibre-optic transillumination, optical caries detection, and quantitative laser and light-induced fluorescence. Advanced techniques like micro-computed tomography (micro-CT) provide detailed, non-invasive 3D imaging, allowing for accurate detection and quantification of lesions [14].

Calcium sucrose phosphate (CaSP) combines calcium and phosphate in a readily soluble form. It is said to contain 11.5% calcium content, which aids in diminishing enamel's susceptibility to acid dissolution and expedites remineralization through a shared ion effect [5].

On the other hand, a compound of casein phosphopeptides and amorphous calcium phosphate (CPP-ACP) can stabilise calcium and phosphate because of its specific amino acid sequence [15,16]. In the past, there have been studies existing in the literature evaluating the efficacy of both CPP-ACP and CaSP in remineralization [17,18]. However, studies pertaining to the three-dimensional analysis of remineralization are still relatively few. This study aims to evaluate and compare the three-dimensional remineralization efficacy of two commercial toothpastes, one of CaSP (Enafix, Group Pharmaceuticals., Ltd, Malur, India) and the other, CCP-ACP (GF Tooth Mousse, GC India Dental Pvt. Ltd., Patancheru, India) on artificially created WSLs through micro-CT assessment.

## Materials and methods

The study comprised freshly removed human premolars free of carious lesions and in good morphological condition. Any teeth that had fractures, breaks, or hypocalcifications were not included. Up to the experiment day, the freshly removed teeth were kept in a saline solution containing 0.1% thymol at a temperature of +4 °C [9,19]. Any residual tissue on the tooth surfaces was removed using an ultrasonic scaler. To create an enamel slab, samples in the three groups were demineralized by immersing them in a demineralisation solution for 72 hours. To achieve this, each group was submerged in 15 millilitres of demineralising solution, which was replaced every 24 hours. The samples were then cleaned using deionized water.

**Figure 1:**
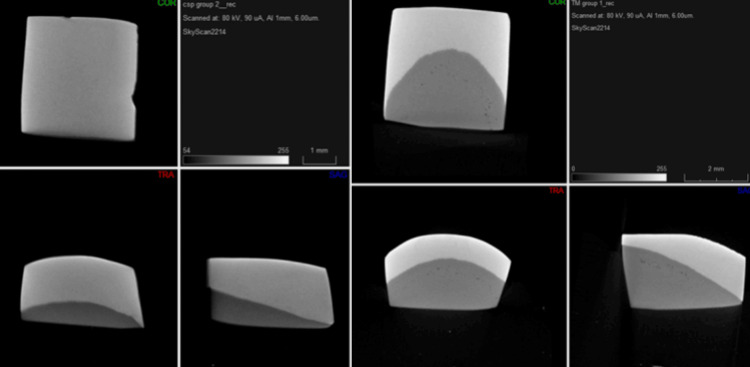
Micro-CT images in the X-axis, Y-axis and Z-axis of the remineralized enamel structure after treatment with CaSP and CPP-ACP scanned at 80 kVp and 90 microA through an aluminium filter of 1mm and a voxel size of 6.00 um. CaSP: calcium sucrose phosphate; CPP-ACP: casein phosphopeptide-amorphous calcium phosphate

The remineralisation solution was refrigerated, and its pH was constantly monitored to ensure efficacy throughout the experimental period. The remineralization cycle in Group 1 involved immersion in 30 ml of remineralizing solution, which was replaced every 24 hours. In the experimental groups, it involved the once-daily application of the experimental toothpaste (Group 2: CaSP and Group 3: CPP-ACP) for a period of five minutes, followed by immersing all the tooth samples in remineralizing solutions (30 ml each). This cycle was continued for a period of 13 days. Following this, the samples were washed with deionized water, and further analysis was done (Figure 1).

Micro-CT

To scan the specimens, hydroxyapatite phantoms weighing 0.25 and 0.75 g/cm3 were utilised as a guide. In an earlier study, a specially made polystyrene container kept the specimens and phantoms steady when they were placed within the cylindrical receiver for the micro-CT scan [20,21]. In the present study, these procedures were followed. The scanning settings were comparable to those found in previously published research on micro-CT scans of specimens made of enamel [22, 23].

The micro-CT machine settings were as follows: Scanner: SkyScan2214 (Bruker Corp., Billerica, MA), Instrument S/N: 20C18034, Software Version: 1.8, Number of Files: 1801, Image Pixel Size (µm): 2.250152, Source Voltage (kV): 80, Source Current (µA): 90, Exposure (ms): 1000, Rotation Step (deg): 0.200, Use 360 Rotation: YES. The filter used was Al 1mm (Figure 2).

Micro-CT values were recorded for all three groups both before and after remineralization. The pre- and post-remineralization values within each group were compared using a paired T-test. Statistical analysis was performed using SPSS for Windows v.19.0 (IBM Corp., Armonk, NY) with the Shapiro-Wilk test employed to assess normality. One-way ANOVA and post-hoc Tukey's HSD tests were used to evaluate statistical significance between the groups, with a significance level set at 0.05.

## Results

The table compares enamel volume and density across different groups subjected to demineralization (Demin), a control group, and two treatment groups (CaSP and CCP-ACP). The findings reveal that no significant difference was observed in enamel volume between demineralized enamel and the control group (p = 0.842), CaSP-treated group (p = 0.472), or CCP-ACP-treated group (p = 0.198). Similarly, no significant difference in the enamel density was found between the demineralized enamel and the control group (p = 0.483), CaSP-treated group (P = 0.811), or CCP-ACP-treated group (p = 0.999) (Table 1).

**Table 1:** Paired sample T-test comparing pre- and post-remineralisation values within the three groups. *A p-value of <0.05 was considered statistically significant. Demin: demineralization only; CaSP: calcium sucrose phosphate; CCP-ACP: casein phosphopeptide-amorphous calcium phosphate; SD: standard deviation

Parameters	Pair	Group	Mean ± SD	P value
Enamel volume	Pair 1	Demin	11.44±1.05	0.842
		Control	15.02±0.40	
	Pair 2	Demin	11.44±1.05	0.472
		CaSP	16.30±1.12	
	Pair 3	Demin	11.44±1.05	0.198
		CCP-ACP	15.26±0.93	
Enamel density	Pair 4	Demin	49.93±5.83	0.483
		Control	57.31±5.88	
	Pair 5	Demin	49.93±5.84	0.811
		CaSP	56.10±5.10	
	Pair 6	Demin	49.93±5.83	0.999
		CCP-ACP	62.43±3.14	

A statistically significant difference (p< 0.01) in the enamel volume and enamel density was found between the groups using a one-way ANOVA test (p = 0.004) (Table 2).

**Table 2:** One-way ANOVA test comparing enamel volume and enamel mineral density *A p-value of <0.05 was considered statistically significant.

Parameter	Groups	Sum of Squares	df	Mean Square	F	P-value
Enamel volume	Between groups	80.743	3	26.914	31.700	<0.01*
	Within groups	16.980	20	.849		
	Total	97.724	23	-		
Enamel mineral density	Between groups	474.650	3	158.217	6.045	0.004*
	Within groups	523.454	20	26.173		
	Total	998.104	23	-		

For enamel volume, the CaSP group had significantly lower values compared to the control group (mean difference = -1.27983, p = 0.026), while the CCP-ACP group did not show a significant difference when compared to either the control or CaSP groups. Regarding enamel mineral density, there was no significant difference between the control and CaSP groups (p = 0.686), but the CCP-ACP group showed a significant improvement compared to CaSP (mean difference = 6.33233, p = 0.045). Additionally, while CCP-ACP had a higher mineral density than the control (mean difference = 5.11876), this difference was not statistically significant (p = 0.098) (Table 3).

**Table 3:** Post hoc Tukey test of the intergroup comparison of the enamel changes *A p-value of <0.05 was considered statistically significant. CaSP: calcium sucrose phosphate; CCP-ACP: casein phosphopeptide-amorphous calcium phosphate

Parameters	Group (I)	Group (J)	Mean Difference (I-J)	Standard Error	P-value
Enamel volume	Control	CaSP	-1.27983^*^	0.53198	0.026*
		CCP-ACP	-.24167	0.53198	0.655
	CaSP	Control	1.27983^*^	0.53198	0.026*
		CCP-ACP	1.03816	0.53198	0.065
	CCP- ACP	Control	.24167	0.53198	0.655
		CaSP	-1.03816	0.53198	0.065
Enamel mineral density	Control	CaSP	1.21357	2.95368	0.686
		CCP-ACP	-5.11876	2.95368	0.098
	CaSP	Control	-1.21357	2.95368	0.686
		CCP-ACP	-6.33233^*^	2.95368	0.045*
	CCP- ACP	Control	5.11876	2.95368	0.098
		CaSP	6.33233^*^	2.95368	0.045*

## Discussion

WSLs often emerge in the central area of a tooth's surface, displaying a noticeable boundary [24]. The shift in oral pathogenic species is one main reason attributed to the occurrence of WSL [25]. Prevention of WSLs among orthodontic patients involves the establishment of a robust oral hygiene routine, which includes usage of toothpaste, mouthwash and varnish-containing remineralizing agents [26]. Despite numerous proposed strategies for preventing and addressing initial enamel lesions, a consensus has yet to be reached regarding the most effective approach to this common problem. In this work, tooth slabs were immersed in a demineralizing solution for 72 hours in order to develop artificial enamel lesions. Following this, the remineralization efficacy of two different toothpastes (CaSP and CPP-ACP) was evaluated using micro-CT assessment. Results of the study revealed that enamel volume changes were better seen with CaSP, and enamel mineral density was better noted with the CCP-ACP toothpaste. This is in accordance with a study where notable enhancements in the microhardness values enamel were found following the application of CaSP in comparison to CPP-ACP, CPP-ACPF, and other remineralizing agents [27].

Additionally, Chen et al.'s study demonstrated the efficacy of several remineralizing agents in treating WSLs during orthodontic treatment, and it was shown that MI Paste Plus, CPP-ACP, and NaF were beneficial in curing enamel lesions [28]. In a previous study, surface enamel characteristics showed that CPP-ACP showed better remineralizing efficiency when compared with CPP-ACPF [28]. Along similar lines, research conducted by Pai et al. showed that topical administration of CPP-ACP resulted in remineralization of the enamel surface [29,30]. Each remineralization agent operates through specific pathways, such as promoting calcium and phosphate ion uptake, enhancing fluoride incorporation, or forming protective layers on enamel surfaces. These varying mechanisms influence the effectiveness of each agent in remineralizing WSLs.

CPP-ACP and CaSP operate through different mechanisms; CPP-ACP stabilizes and releases ions for enamel remineralization, while CaSP releases ions that form a protective apatite layer. Despite these differences, the results showed comparable effectiveness in promoting remineralization and protecting enamel. In the current study, both toothpaste formulations were found to have clinically similar efficacy in the management of artificial enamel lesions. Several studies have already analysed the clinical efficacy of CPP-ACP paste. However, considering the cost factor, CaSP can be considered a suitable alternative. Clinical studies with the same can be done to identify the clinical performance of the CaSP paste.

Limitations

One limitation of the current study is that it is an in vitro study, not a clinical one, conducted under experimental conditions with a limited sample size. This means that the results need to be validated with clinical outcome analysis in the oral environment.

## Conclusions

Based on the limitations within the scope of the present inquiry, it appears that CaSP demonstrates comparable efficacy to CPP-ACP in the remineralization of artificial enamel lesions. While further research may be warranted to validate these findings across broader contexts and diverse populations, the current evidence suggests a potential equivalence between these two agents in promoting enamel remineralization. These findings contribute to our understanding of alternative strategies for dental care and highlight the potential of CaSP as a viable remineralizing agent alongside CPP-ACP.

## References

[1] Karad A, Dhole P, Juvvadi S, Joshi S, Gupta A (2019). White spot lesions in orthodontic patients: An expert opinion. J Int Oral Health.

[2] Featherstone JD (2008). Dental caries: a dynamic disease process. Aust Dent J.

[3] Karad A (2014). Clinical Orthodontics: Current Concepts, Goals and Mechanics. https://shop.elsevier.com/books/clinical-orthodontics-current-concepts-goals-and-mechanics/karad/978-81-312-3739-7.

[4] Mizrahi E (1982). Enamel demineralization following orthodontic treatment. Am J Orthod.

[5] Menon LU, Varma RB, Kumaran P, Xavier AM, Govinda BS, Kumar JS (2018). Efficacy of a calcium sucrose phosphate based toothpaste in elevating the level of calcium, phosphate ions in saliva and reducing plaque: a clinical trial. Contemp Clin Dent.

[6] Malcangi G, Patano A, Morolla R (2023). Analysis of dental enamel remineralization: a systematic review of technique comparisons. Bioengineering (Basel).

[7] Solanki LA, Shantha Sundari KK, Muralidharan NP, Jain RK (2022). Antimicrobial effect of novel gold nanoparticle oral rinse in subjects undergoing orthodontic treatment: an ex-vivo study. J Int Oral Health.

[8] Deepika BA, Ramamurthy J, Girija S, Jayakumar ND (2022). Evaluation of the antimicrobial effect of Ocimum sanctum L. oral gel against anaerobic oral microbes: An in vitro study. World J Dent.

[9] Khoroushi M, Kachuie M (2017). Prevention and treatment of white spot lesions in orthodontic patients. Contemp Clin Dent.

[10] Ramya G, Rajasekar A (2021). Enhanced antibacterial effect of titanium dioxide nanoparticles mediated grape seed extract on oral pathogens - streptococcus mutans and lactobacillus. J Evol Med Dent Sci.

[11] Verma P, Muthuswamy Pandian S (2021). Bionic effects of nano hydroxyapatite dentifrice on demineralised surface of enamel post orthodontic debonding: in-vivo split mouth study. Prog Orthod.

[12] Weyland MI, Jost-Brinkmann PG, Bartzela T (2022). Management of white spot lesions induced during orthodontic treatment with multibracket appliance: a national-based survey. Clin Oral Investig.

[13] Verma V, Jain RK (2022). Visual assessment of extent of white spot lesions in subjects treated with fixed orthodontic appliances: a retrospective study. World J Dent.

[14] Akküç S, Duruk G, Keleş A (2023). Remineralization effect of three different agents on initial caries and erosive lesions: a micro-computed tomography and scanning electron microscopy analysis. BMC Oral Health.

[15] Grewal N, Kudupudi V, Grewal S (2013). Surface remineralization potential of casein phosphopeptide-amorphous calcium phosphate on enamel eroded by cola-drinks: An in-situ model study. Contemp Clin Dent.

[16] Gurunathan D, Somasundaram S, Kumar S (2012). Casein phosphopeptide-amorphous calcium phosphate: a remineralizing agent of enamel. Aust Dent J.

[17] Oliveira GM, Ritter AV, Heymann HO, Swift E Jr, Donovan T, Brock G, Wright T (2014). Remineralization effect of CPP-ACP and fluoride for white spot lesions in vitro. J Dent.

[18] Vinod D, Gopalakrishnan A, Subramani SM, Balachandran M, Manoharan V, Joy A (2020). A comparative evaluation of remineralizing potential of three commercially available remineralizing agents: an in vitro study. Int J Clin Pediatr Dent.

[19] Kamath P, Nayak R, Kamath SU, Pai D (2017). A comparative evaluation of the remineralization potential of three commercially available remineralizing agents on white spot lesions in primary teeth: An in vitro study. J Indian Soc Pedod Prev Dent.

[20] Stookey GK, Featherstone JD, Rapozo-Hilo M (2011). The Featherstone laboratory pH cycling model: a prospective, multi-site validation exercise. Am J Dent.

[21] Argenta RM, Tabchoury CP, Cury JA (2003). A modified pH-cycling model to evaluate fluoride effect on enamel demineralization. Pesqui Odontol Bras.

[22] Bijle MN, Ekambaram M, Lo EC, Yiu CK (2020). The enamel remineralization potential of fluoride varnishes containing arginine. J Dent.

[23] Bijle MN, Abdalla MM, Ashraf U, Ekambaram M, Yiu CK (2021). Enamel remineralization potential of arginine-fluoride varnish in a multi-species bacterial pH-cycling model. J Dent.

[24] Anil AI, Ibraheem WI, Meshni AA, Preethanath R, Anil S (2022). Demineralization and remineralization dynamics and dental caries. Dental Caries - The Selection of Restoration Methods and Restorative Materials.

[25] Song Z, Fang S, Guo T, Wen Y, Liu Q, Jin Z (2023). Microbiome and metabolome associated with white spot lesions in patients treated with clear aligners. Front Cell Infect Microbiol.

[26] Bishara SE, Ostby AW (2008). White spot lesions: formation, prevention, and treatment. Semin Orthod.

[27] Karad A; Dhole P (2019). Evaluation of remineralizing efficacy of calcium sucrose phosphate: A systematic review of in vitro studies. J Indian Orthod Soc.

[28] Chen H, Liu X, Dai J, Jiang Z, Guo T, Ding Y (2013). Effect of remineralizing agents on white spot lesions after orthodontic treatment: a systematic review. Am J Orthod Dentofacial Orthop.

[29] Jayarajan J, Janardhanam P, Jayakumar P (2011). Efficacy of CPP-ACP and CPP-ACPF on enamel remineralization - an in vitro study using scanning electron microscope and DIAGNOdent. Indian J Dent Res.

[30] Rajendran R, Hussain MS, Sandhya R, Ashik M, Thomas AJ, Mammen RE (2022). Effect of remineralization agents on white spot lesions: a systematic review. J Pharm Bioallied Sci.

